# Cardiac filling volumes versus pressures for predicting fluid responsiveness after cardiovascular surgery: the role of systolic cardiac function

**DOI:** 10.1186/cc10062

**Published:** 2011-02-25

**Authors:** Ronald J Trof, Ibrahim Danad, Mikel WL Reilingh, Rose-Marieke BGE Breukers, AB Johan Groeneveld

**Affiliations:** 1 Vrije Universiteit Medical Centre, Department of Intensive Care, De Boelelaan 1117, 1081 HV Amsterdam, The Netherlands; 2 Medisch Spectrum Twente, Department of Intensive Care, Haaksbergerstraat 55, 7513 ER Enschede, The Netherlands

## Abstract

**Introduction:**

Static cardiac filling volumes have been suggested to better predict fluid responsiveness than filling pressures, but this may not apply to hearts with systolic dysfunction and dilatation. We evaluated the relative value of cardiac filling volume and pressures for predicting and monitoring fluid responsiveness, according to systolic cardiac function, estimated by global ejection fraction (GEF, normal 25 to 35%) from transpulmonary thermodilution.

**Methods:**

We studied hypovolemic, mechanically ventilated patients after coronary (*n *= 18) or major vascular (*n *= 14) surgery in the intensive care unit. We evaluated 96 colloid fluid loading events (200 to 600 mL given in three consecutive 30-minute intervals, guided by increases in filling pressures), divided into groups of responding events (fluid responsiveness) and non-responding events, in patients with low GEF (<20%) or near-normal GEF (≥20%). Patients were monitored by transpulmonary dilution and central venous (*n *= 9)/pulmonary artery (*n *= 23) catheters to obtain cardiac index (CI), global end-diastolic volume index (GEDVI), central venous (CVP) and pulmonary artery occlusion pressure (PAOP).

**Results:**

Fluid responsiveness occurred in 8 (≥15% increase in CI) and 17 (≥10% increase in CI) of 36 fluid loading events when GEF was <20%, and 7 (≥15% increase in CI) and 17 (≥10% increase in CI) of 60 fluid loading events when GEF was ≥20%. Whereas a low baseline GEDVI predicted fluid responsiveness particularly when GEF was ≥20% (*P *= 0.002 or lower), a low PAOP was of predictive value particularly when GEF was <20% (*P *= 0.004 or lower). The baseline CVP was lower in responding events regardless of GEF. Changes in CVP and PAOP paralleled changes in CI particularly when GEF was <20%, whereas changes in GEDVI paralleled CI regardless of GEF.

**Conclusions:**

Regardless of GEF, CVP may be useful for predicting fluid responsiveness in patients after coronary and major vascular surgery provided that positive end-expiratory pressure is low. When GEF is low (<20%), PAOP is more useful than GEDVI for predicting fluid responsiveness, but when GEF is near-normal (≥20%) GEDVI is more useful than PAOP. This favors predicting and monitoring fluid responsiveness by pulmonary artery catheter-derived filling pressures in surgical patients with systolic left ventricular dysfunction and by transpulmonary thermodilution-derived GEDVI when systolic left ventricular function is relatively normal.

## Introduction

The clinical benefit of various hemodynamic monitoring techniques in the critically ill is still under debate [1-5]. Static filling volumes, such as the transpulmonary dilution-derived global end-diastolic volume, have been suggested to better predict fluid responsiveness than filling pressures such as the central venous pressure (CVP) or pulmonary artery occlusion pressure (PAOP) obtained from a pulmonary artery catheter [6-19]. Most studies, however, often included patients with relatively normal left ventricular systolic function, undergoing coronary artery surgery [6-13,15,16,19]. Mundigler *et al. *suggested that pressures were superior to transpulmonary thermodilution-derived volumes for monitoring changes in cardiac preload during fluid loading in non-surgical patients with left ventricular systolic dysfunction, measured by transesophageal echocardiography [20]. We also suggested this in patients with presumed left ventricular systolic dysfunction based on transpulmonary thermodilution-derived global ejection fraction (GEF) following valvular surgery [21]. However, others did not reach the same conclusion [14,17]. Nevertheless, according to Laplace's Law, pressures and volumes may both contribute to end-diastolic wall stress as a true measure of cardiac preload. Based on the curvilinear left ventricular pressure-volume relationship at end-diastole, volumes may increase more than pressures with fluid loading at low cardiac filling, while at higher cardiac filling, pressures may increase more than volumes [5]. At low cardiac filling, volumes may thus better predict fluid responsiveness than pressures, while in hearts with systolic dysfunction and dilatation, pressures may better predict and monitor fluid responsiveness than volumes [5,22].

We hypothesized that during fluid loading in patients with reduced systolic cardiac function as compared to those with normal function, filling pressures may be superior to filling volumes (that is, global end-diastolic volume, GEDV) for predicting and monitoring of fluid responsiveness, and vice versa. We thus measured prospectively cardiac filling pressures and volumes in hypovolemic patients following cardiovascular and major vascular surgery, using the pulmonary artery catheter and transpulmonary thermodilution technique, prior to, during and following colloid fluid loading.

## Materials and methods

This is a substudy of a prospective, non-randomized, single-center clinical trial, investigating the volume expanding effects of various resuscitation fluids [23]. The study was approved by the Ethics Committee of the Vrije Universiteit Medical Center. Written informed consent was obtained pre-operatively. We analyzed the effect of colloid fluid loading in patients who had undergone coronary artery (*n *= 18) or major vascular surgery (*n *= 14). Colloid fluid loading was given with modified fluid gelatin 4%, hydroxyethyl starch (HES) 6% or albumin 5%, all of which have similar oncotic properties and hemodynamic responses [23]. We only analyzed patients who completed fluid loading and measurements up to t = 90 minutes. Inclusion criteria, at enrollment and start of the protocol, were presumed hypovolemia, defined as a systolic blood pressure <110 mmHg and reduced filling pressures: PAOP <13 mmHg (in the presence of a pulmonary artery catheter) or CVP <12 mmHg. Exclusion criteria were age >75 year, preterminal illness with a life expectancy of less than 24 hours, or known anaphylactic reactions to colloids. All peri-operative care was given by attending physicians who were not involved in the study.

### Study protocol

The protocol was started upon arrival of the patients in the intensive care unit (ICU). Demographic characteristics were recorded, including the Acute Physiology And Chronic Health Evaluation II (APACHE-II) score and transesophageal echocardiographic findings prior to surgery. At baseline (t = 0 minute), hemodynamic measurements were performed. Heart rate (HR) and mean arterial pressure (MAP) from a radial artery were recorded at t = 0 and 90 minutes. The HR was taken from the continuously recorded electrocardiogram. The mean pulmonary artery pressure (MPAP) was measured at t = 0 and 90 minutes. Cardiac output, GEDV, CVP and PAOP were measured every 30 minutes, from t = 0 to 90 minutes. Pressures were measured with patients in the supine position after calibration, zeroing to atmospheric pressure and, for PAOP, after proper wedging, at the midchest level at end-expiration (Tramscope^®^, Marquette, GE, Milwaukee, WI, USA). For the measurements of cardiac output and GEDV, the transpulmonary thermal-dye indicator dilution technique was used [1,6]. These measurements involve a central venous injection of 15 mL of ice-cold indocyanine green in 5% glucose solution and concomitant registration of the dilution curves in the femoral artery, by a 3F catheter equipped with a thermistor (PV 2024, Pulsion Medical Systems, Munich, Germany). This catheter was inserted at the end of surgery via a 4F introducing sheath (Arrow International, Inc., Reading, PA, USA) and connected to a bedside computer (COLD Z-021, Pulsion Medical Systems, Munich, Germany. The COLD Z-021 is the precursor to the current pulse contour cardiac output (PiCCO™) technique. GEDV represents the volumes of the right and left heart at end-diastole and reflects left ventricular dimensions obtained by echocardiography in the absence of overt right ventricular distention [7,12-17]. The ratio between stroke volume and global end-diastolic volume/4 is defined as the global ejection fraction (GEF, normal values 25 to 35%), and is an indicator of left ventricular systolic function, provided that there is no right ventricular dysfunction [24,25]. Reproducibility of these measurements is typically within 10% [1]. After baseline measurements were taken, fluids were given over 90 minutes on the basis of the response within predefined limits of increases in pressures (CVP or - when available - PAOP), according to a previously described protocol [23,26,27]. Up to 200 mL of fluid were given every 10 minutes, provided that the increase in filling pressures with the fluid loading did not exceed critical values, and this policy has been proven safe in previous studies (that is, not evoking pulmonary edema) [23,26,27]. The maximum amount of fluid infused was 1,800 mL. Concomitant vasoactive and sedative drug treatment and ventilatory settings remained unchanged during fluid loading. Indeed, all patients received volume-controlled mechanical ventilation and positive end-expiratory pressure (PEEP). Drainage of blood was <50 mL/hour in all patients, and no patient underwent repeated surgery for bleeding within 12 hours post surgery.

### Statistical analysis

The groups to be analyzed were divided into low GEF (<20%) and near-normal GEF (≥20%). The cutoff of 20% approximately reflects a cutoff of 40% ejection fraction of the left ventricle, the lower limit of normal, as measured by echocardiography, provided that there is no right ventricular dysfunction [24,25]. We also analyzed data according to a cutoff of 15%. Stroke volume, cardiac output and global end-diastolic volume were indexed to body surface area (BSA), giving stroke volume index (SVI, mL/m^2^), cardiac index (CI, L/minute/m^2^) and global end-diastolic volume index (GEDVI, n 680 to 800 mL/m^2^), respectively. Cardiac distensibility was determined as the surrogate for cardiac compliance and was calculated by GEDVI/(CVP + PAOP)/2 (mL/m^2^/mmHg), or GEDVI/CVP if PAOP was not available [28]. Fluid responsiveness was defined as an increase of CI or SVI ≥10% or ≥15%, in accordance with the literature [4,9,17], between t = 0 to 30, t = 30 to 60 and t = 60 to 90 minutes during fluid loading. For categorical data, X^2 ^and Fisher exact tests were used. Since continuous data were normally distributed (Kolmogorov-Smirnov test, *P *> 0.05), they were summarized by mean ± standard deviation (SD) and parametric tests were done. Paired and unpaired t-tests were used to compare data in time and between GEF groups (for Table 1). Generalized estimating equations (GEE) were used to evaluate differences in baseline and changes in variables between summated responding and non-responding fluid loading events in each GEF group (for Table 2), to evaluate their predictive and monitoring values, respectively, taking repeated measurements in the same patients into account, with the amount and type of fluid infused entered as covariates to adjust for potential confounding. Partial correlation coefficients (r), adjusted for repeated measurements by entering patient number and for type and amount of fluids as covariates were calculated. Coefficients were compared after z transformation. Receiver operating characteristic curves (ROC) plotting sensitivity against 1-specificity were constructed to evaluate the predictors of fluid responsiveness by the areas under the curve (AUC, with 95% confidence intervals) for pooled data, in the absence of accepted methods to adjust for repeated measurements, and were compared with each other. Optimum cutoff values with associated combinations of highest sensitivity and specificity were calculated (MedCalc Software, Mariakerke, Belgium). Exact two-sided *P*-values > 0.001 are given and considered statistically significant when <0.05. All analyses were conducted using SPSS version 15.0 (SPSS Inc, Chicago, Ill, USA).

**Table 1:** Patient characteristics

	GEF <20% (*n *= 12)	GEF ≥20% (*n *= 20)	*P*-value
**Demographic variables**			
Age	66 ± 7	61 ± 7	0.082
Male/female	9/3	16/4	1.000
APACHE II	9 ± 4	9 ± 3	0.690
Coronary artery/major vascular surgery	5/7	13/7	0.2777
CPB yes/no	4/1	9/4	0.648
time of CPB, minute	97 ± 72	78 ± 58	0.564
Echocardiography (LVEF before surgery) good (≥40%)/poor (<40%)	3/9	16/4	1.000
**Hemodynamic and respiratory variables**			
HR, b/minute			
T = 0	75 ± 11	68 ± 12	0.112
T = 90	72 ± 12	72 ± 14^1^	0.101 (for increase)
MAP, mmHg			
T = 0	85 ± 15	74 ± 12	0.034
T = 90	92 ± 19	84 ± 10^2^	0.608 (for increase)
CVP, mmHg			
T = 0	5 ± 2	3 ± 2	0.047
T = 30	7 ± 3	5 ± 2	n.a.
T = 60	8 ± 3	6 ± 2	n.a.
T = 90	8 ± 2^3^	7 ± 2^3^	0.813 (for increase)
MPAP, mmHg			
T = 0	17 ± 6	15 ± 4	0.260
T = 90	2 ± 35^3^	21 ± 4^3^	0.627 (for increase)
PAOP, mmHg			
T = 0	6 ± 3	7 ± 3	0.477
T = 30	9 ± 2	9 ± 2	n.a.
T = 60	11 ± 3	10 ± 3	n.a.
T = 90	12 ± 2^3^	11 ± 2^3^	0.037 (for increase)
GEDVI, mL/m^2^			
T = 0	1,049 ± 247	830 ± 195	0.009
T = 30	1,132 ± 360	840 ± 174	n.a.
T = 60	1,170 ± 387	857 ± 171	n.a.
T = 90	1,220 ± 476	861 ± 189	0.089 (for increase)
SVI, mL/m^2^			
T = 0	42 ± 10	52 ± 12	0.022
T = 90	47 ± 9^4^	56 ± 14^5^	0.030 (for increase)
CI, mL/m^2^			
T = 0	3.1 ± 0.7	3.4 ± 0.6	0.170
T = 30	3.5 ± 0.7	3.7 ± 0.7	n.a.
T = 60	3.7 ± 0.9	3.9 ± 0.8	n.a.
T = 90	3.9 ± 0.9^3^	3.9 ± 0.6^3^	0.101 (for increase)
GEF, %			
T = 0	16 ± 4	25 ± 5	n.a.
T = 90	19 ± 3	26 ± 4	n.a.
Distensibility, mL/m^2^/mmHg			
T = 0	241 ± 167	229 ± 124	0.830
T = 90	132 ± 64^1^	124 ± 60^2^	0.910 (for decrease)
PEEP, cmH_2_O			
T = 0	7.5 ± 2.0	6.7 ± 2.7	0.385
Fluid infused, mL	1,466 ± 296	1,585 ± 291	0.300
Gelatin/HES/albumin	2/3/7	5/8/7	0.436
Fluid balance, mL	1,001 ± 334	1,034 ± 497	0.839

Mean ± SD. APACHE II, Acute Physiology And Chronic Health Evaluation II; CI, cardiac index; CPB, cardiopulmonary bypass; CVP, central venous pressure; GEDVI, global end-diastolic volume index; GEF, global ejection fraction; HES, hydroxyethyl starch; HR, heart rate; LV, left ventricular; LVEF, left ventricular ejection fraction; MAP, mean arterial pressure; MPAP, mean pulmonary artery pressure; PAOP, pulmonary artery occlusion pressure; PEEP, positive end-expiratory pressure. t = 0 and 90 min: prior to and at completion of fluid loading; ^1^*P *< 0.05; ^2^*P *= 0.001; ^3^*P *< 0.001; ^4^*P *= 0.017; ^5^*P *= 0.007 vs. t = 0; n.a., not applicable.

**Table 2:** Summated fluid loading responsiveness (≥10% increase in cardiac index) when global ejection fraction is <20% or ≥20%

	GEF <20% (*n *= 12)			GEF ≥ 20% (*n *= 20)		
	Responder (*n *= 17 steps in 10 patients)	Non-responder (*n *= 19 steps in 11 patients)	*P*-value	Responder (*n *= 17 steps in 14 patients)	Non-responder (*n *= 43 steps in 20 patients)	*P*-value
						
CI, L/minute/m^2^						
baseline	3.3 ± 0.9	3.6 ± 0.8	0.095	3.3 ± 0.5	3.8 ± 0.8	0.028
after	3.9 ± 0.8	3.6 ± 0.8		3.9 ± 0.7	3.8 ± 0.7	
change	0.6 ± 0.1	0.0 ± 0.1	n.a.	0.6 ± 0.6	0.0 ± 0.3	n.a.
						
GEDVI, mL/m^2^						
baseline	1,123 ± 422	1,111 ± 234	0.506	754 ± 176	877 ± 167	0.011
after	1,254 ± 518	1,102 ± 246		812 ± 163	869 ± 179	
change	130 ± 175	-8 ± 73	<0.001	58 ± 63	-8 ± 62	0.003
						
CVP, mmHg						
baseline	5 ± 3	8 ± 3	0.004	3 ± 2	5 ± 2	0.027
After	6 ± 2	9 ± 2		5 ± 2	6 ± 2	
change	1 ± 1	1 ± 2	0.013	1 ± 1	1 ± 1	0.468
						
PAOP, mmHg						
baseline	8 ± 3	11 ± 3	0.003	8 ± 2	9 ± 3	0.150
after	10 ± 2	13 ± 4		10 ± 3	11 ± 3	
change	2 ± 1	1 ± 2	0.083	1 ± 1	1 ± 2	0.563
						
Fluid input per step, mL	541 ± 100	442 ± 135	0.019	541 ± 123	523 ± 113	0.377

Mean ± SD.

CI, cardiac index; CVP, central venous pressure; GEDVI, global end diastolic volume index; GEF, global ejection fraction; n.a., not applicable; NR, non-responding fluid loading step; PAOP, pulmonary artery occlusion pressure: *n *= 13, *n *= 10, *n *= 11 and *n *= 21, in R and NR at GEF <20% and ≥20%, respectively; R, responding fluid loading step (≥10% increase in CI). *P-*values adjusted for amount and type of fluid.

## Results

Table 1 summarizes the demographic, hemodynamic and respiratory characteristics of patients. Patients underwent coronary artery or major vascular surgery (in three cases on the distal thoracic aorta). Surgery was uneventful in all patients. The table shows the differences between patient groups with a GEF <20% and ≥20% and the changes with fluid loading. There was no difference in the amount and type of fluids infused and fluid balances between the GEF groups. GEF did not change during fluid loading. Baseline GEDVI was higher when GEF was <20% than ≥20% suggesting cardiac dilatation. Preoperative echocardiography did not document severe right ventricular dysfunction and dilatation in any patient. There was no postoperative pulmonary hypertension and MPAP was 28 mmHg at maximum in one patient. Indeed, MPAP at t = 90 minutes in the low GEF group was 23 ± 7 and 25 ± 2 mmHg and in the near-normal GEF group 21 ± 4 and 22 ± 4 mmHg, in responders and non-responders, respectively (GEE: *P *= 0.44 for response, *P *= 0.99 for GEF). Similarly, the MAP at t = 90 minutes in the low GEF group was 95 ± 16 and 86 ± 25 mmHg and in the near-normal GEF group 83 ± 7 and 85 ± 12 mmHg, in responders and non-responders, respectively (GEE: *P *= 0.52 for response, *P *= 0.98 for GEF).

### Fluid loading events

Among the 96 fluid loading events, the proportion of responding events (increase in CI ≥10%) decreased from t = 0 to 90 minutes (*P *= 0.031). The amount infused was somewhat lower in non-responding than in responding events when GEF was low (<20%), (Table 2). Baseline CI was lower in responding events, regardless of GEF and cutoff percentage of fluid responsiveness. When GEF was low, baseline CVP and PAOP were lower for responding events (≥10% increase in CI) while baseline GEDVI did not differ from that in non-responding events, irrespective of the amount and type of fluids. When GEF was near-normal (≥20%), baseline GEDVI and CVP were lower for responding events (≥10% increase in CI), while baseline PAOP did not differ from that in non-responding events. Similar results were obtained for a GEF cutoff of 15% (Table S1 in Additional file 1).

For fluid responsiveness defined as an increase in CI ≥15%: only baseline PAOP and not CVP predicted fluid responsiveness in the low GEF group (Table S2 in Additional file 1). In contrast, GEDVI particularly predicted fluid responsiveness when GEF was near-normal. Changes in GEDVI paralleled CI responses in both GEF groups, while changes in CVP paralleled CI responses only in the low GEF group. Changes in PAOP particularly paralleled responses in CI when GEF was low.

### Correlations

**Figure 1:**
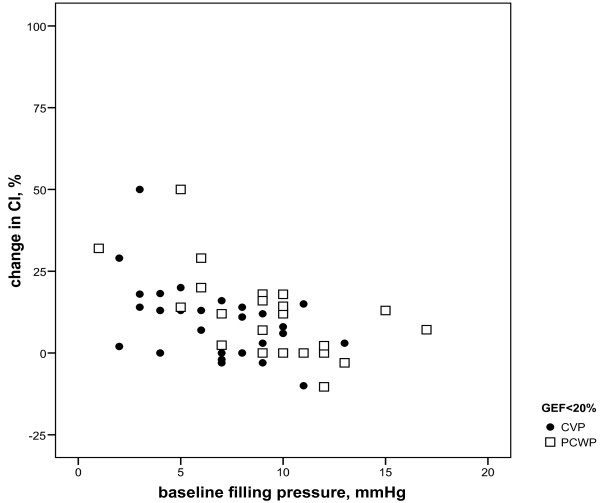
**Baseline filling pressures (PAOP, CVP) versus change in cardiac index (CI) when global ejection fraction (GEF) is low (<20%): r = -0.57, *P *= 0.008 and r = -0.44, *P *= 0.010, respectively**.

**Figure 2:**
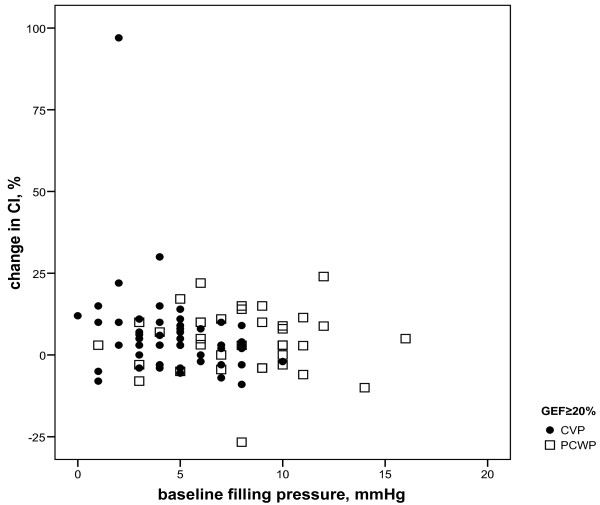
**filling pressures (PAOP, CVP) versus change in cardiac index (CI) when global ejection fraction (GEF) is near-normal (≥20%): r = -0.01, *P *= 0.951 and r = -0.35, *P *= 0.009, respectively Baseline**. For difference between r: *P *= 0.023.

**Figure 3:**
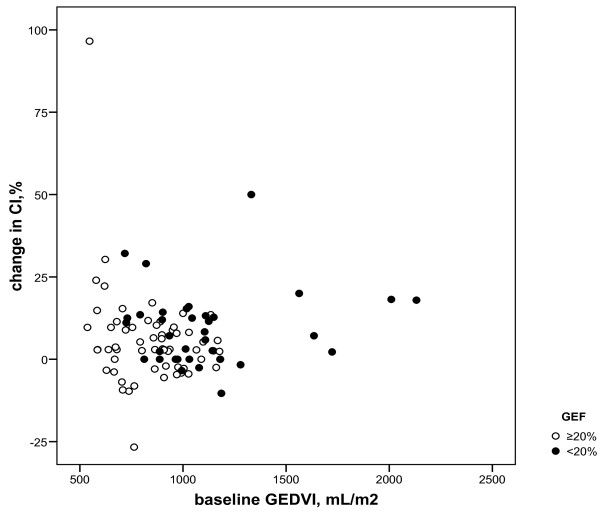
**Baseline global end-diastolic volume (GEDVI) versus change in cardiac index (CI) according to global ejection fraction (GEF)**. In ≥20% GEF group r = -0.29, *P *= 0.03, in <20% GEF group r = 0.17, *P *= 0.33. For difference between r: *P *= 0.048.

For the low GEF group, baseline PAOP and CVP inversely correlated to changes in CI, irrespective of amount and type of fluids (r = -0.57 and -0.44, *P *= 0.008 and 0.010, respectively; Figure 1). In the near-normal GEF group, only baseline CVP inversely correlated to CI changes (r = -0.35, *P *= 0.009) and PAOP did not (Figure 2). Baseline GEDVI inversely correlated to changes in CI in the near-normal GEF group (r = -0.29, *P *= 0.03; Figure 3). Changes in CI were paralleled by changes in GEDVI (r = 0.74, *P *< 0.001) in the low GEF group. Changes in CI correlated to changes in both CVP and GEDVI in the near-normal GEF group (r = 0.36 and r = 0.72, *P *= 0.007 and <0.001, respectively). Changes in PAOP correlated better to CVP in the near-normal GEF group (r = 0.67, *P *< 0.001) than in the low GEF group (r = 0.21, *P *= 0.404).

### Predictors of fluid responsiveness in ROC curves

In the near-normal GEF group, baseline GEDVI and CVP predicted fluid responsiveness (increase in both CI ≥10% and ≥15%), while in the low GEF group baseline PAOP and CVP had predictive value (Table 3). This table also shows the optimum cutoff values and associated sensitivities and specificities for fluid responsiveness. Table S3 in Additional file 1 shows identical results for cutoffs of SVI responses (0 to 90 minutes) rather than of CI responses.

**Table 3:** Areas under the receiver operating characteristic curve (95% confidence intervals) for prediction of fluid responsiveness (increase in cardiac index ≥10% (A) or ≥15% (B)) by baseline values, according to global ejection fraction

	GEF <20% (*n *= 12)			GEF ≥20% (*n *= 20)		
	AUC	*P*-value	Cutoff	AUC	*P*-value	Cutoff
**A**						
GEDVI	0.56 (0.39 to 0.73)	0.511	902	0.72 (0.58 to 0.83)	0.002	890
CVP	0.76 (0.59 to 0.88)	0.001	6	0.73 (0.60 to 0.84)	<0.001	2
PAOP	0.79 (0.57 to 0.93)	0.004	10	0.65 (0.46 to 0.81)	0.129	9
						
**B**						
GEDVI	0.62 (0.44 to 0.77)	0.33	1279	0.89 (0.78 to 0.95)	<0.001	623
CVP	0.77 (0.60 to 0.89)	0.002	5	0.73 (0.60 to 0.84)	0.013	4
PAOP	0.84 (0.63 to 0.96)	<0.001	9	0.50 (0.32 to 0.69)*	0.98*	9

CVP, central venous pressure, mm Hg; GEDVI, global end diastolic volume index, mL/m^2^; GEF, global ejection fraction; PAOP, pulmonary artery occlusion pressure, mm Hg.

*P = 0.008 vs. AUC GEDVI; for A and low GEF: PAOP sensitivity 92%, specificity 60%, positive predictive value 75%, negative predictive value 86%; for normal GEF: GEDVI sensitivity 82%, specificity 56%, positive predictive value 42%, negative predictive value 89%; for B and low GEF: PAOP sensitivity 86%, specificity 69%, positive predictive value 55%, negative predictive value 92%; for normal GEF: GEDVI sensitivity 71%, specificity 94%, positive predictive value 63%, negative predictive value 93%.

## Discussion

Our study suggests that in patients after coronary and major vascular surgery the predictive value of cardiac filling pressures and volumes for fluid responsiveness depends on GEF, as calculated by transpulmonary dilution-derived parameters.

In patients with low GEF indicating systolic cardiac dysfunction, PAOP has a greater predictive value than GEDVI for fluid responsiveness, whereas in patients with near-normal GEF, GEDVI is superior to PAOP. This suggests the increasing value of filling pressures over volumes for predicting fluid responsiveness in patients with left ventricular systolic dysfunction. Indeed, the suggestion that a low GEF reflects systolic dysfunction of the left ventricle is supported by the fact that changes in PAOP did not correlate with changes in CVP, as reported by others [29-31]. Furthermore, our data suggest that PAOP relates to systolic and not to diastolic function since distensibility did not differ between the low and near-normal GEF groups, both prior to and after fluid loading. There was no sign of pulmonary hypertension or difference in MPAP according to fluid responses, thus diminishing the likelihood for right ventricular dysfunction confounding GEDVI as a reflection of left ventricular end-diastolic volume. Hence, the low GEF was likely caused by postoperative left ventricular dysfunction, as the preoperative echocardiographic left ventricular function did not differ among GEF groups. Hence, the greater predictive value of PAOP than of CVP, according to GEF, can be explained by greater effect of left than of right ventricular loading on fluid responsiveness, although we did not directly assess postoperative biventricular function, for instance, by echo. Conversely, the predictive value of CVP for fluid responsiveness regardless of GEF may indicate the importance of venous return for augmenting cardiac output, rather than right ventricular dysfunction following increased afterload limiting a rise in cardiac output with fluids when CVP is relatively high as suggested recently [32]. Finally, the similar course of MAP according to fluid responses disfavors alterations in systemic vascular tone confounding the effect of preload augmentation and its assessment during fluid loading.

The frequency of fluid responsiveness generally agrees with the literature, utilizing various loading protocols, and also involving variable amounts of fluid, in cardiac surgery patients [9,10,12,14-17,19,20]. That both CVP and PAOP were of predictive and monitoring value in our study can be attributed in part to the fact that a relatively low PEEP was applied, so that atmospheric pressure-referenced filling pressures may have approached transmural values. That fluid responsiveness was not uniformly observed in spite of clinical signs of hypovolemia can be attributed to the relatively poor predictive value of the latter, as commonly described [33]. Our study does not address the effect of mathematical coupling of GEDVI to CI, when volumes are derived from the same transpulmonary dilution curve as cardiac output. The often observed superiority of cardiac volumes over filling pressures in predicting and monitoring cardiac output responses, that is, fluid responsiveness, may indeed be overestimated by the phenomenon, as recently described by our group also [1,6-8,10-16,18,19,27]. In hearts with systolic dysfunction and dilatation, a right- and downward shift on the Frank-Starling curve and along the curvilinear pressure-volume relationship at end-diastole, preload recruitability may be more dependent on and thus predicted and monitored by pressures than by volumes [5,22]. Indeed, GEDVI was higher in patients with a low versus a near-normal GEF, suggesting cardiac dilatation. Cardiac distensibility did not differ among GEF groups, favoring a similar position of the diastolic pressure-volume relation and diastolic function. Our data, obtained in surgical patients, thus confirms the Mundigler *et al. *data in non-surgical patients with reduced left ventricular systolic function due to dilated and ischemic cardiomyopathy [20]. The authors showed that in patients with left ventricular systolic dysfunction, the value of transpulmonary thermodilution-derived total end-diastolic volume is particularly insensitive for monitoring the effects of fluid administration on cardiac preload when compared to filling pressures. On the other hand, in patients with normal left ventricular systolic function, volumes and pressures were of equal value [20].

Reuter *et al. *and Preisman *et al. *[14,17] did not observe different monitoring values of filling volumes or pressures according to left ventricular ejection fraction and this can be attributed, in part, to the small number of patients in their studies and their (varying) definitions of left ventricular systolic dysfunction (ejection fraction <35% in the former and <40% in the latter). Nevertheless, the trend was for the increasing value of pressure monitoring in patients with low versus those with normal GEF in the study by Reuter *et al. *[14]. The current data also agree with our previous study in a cohort of valvular and coronary artery surgery patients [21], showing the superior value of the pulmonary artery catheter-derived pressures over transpulmonary dilution-derived volumes for assessing fluid responsiveness in the former with a low GEF and presumed left ventricular systolic dysfunction. The current study thus suggests that systolic cardiac function and the degree of cardiac dilatation, rather than underlying disease (type of surgery), determines the relative value of pressures and volumes for predicting and monitoring fluid responsiveness, as suggested previously [5].

Our study has some limitations. Since our analyses adjusted for amount and type of fluids, it is unlikely that small differences in the amounts of fluids (mean 100 mL when GEF was <20%, for instance) rather than differences in cardiac preloading, were responsible for different increases in CI (of 0.6 L/minute/m^2 ^when GEF was <20%) in responding versus non-responding fluid loading events. The fluid loading protocol guided by changes in filling pressures was used to prevent deleterious fluid overloading rather than to guide treatment on the basis of fluid responsiveness, as recently advocated to ensure safety [23,26,27]. By virtue of its design, the study did not address the potential clinical benefits of one hemodynamic monitoring technique over the other. Although our results were obtained by thermal-dye dilution, the current standard is single transpulmonary thermodilution (PiCCO™ technique) [10,34], because double and single dilution methods yield similar values for GEDVI. Hence, our results should also be applied to single transpulmonary thermodilution. Although dynamic indices (for example, pulse pressure or stroke volume variation) are better predictors of fluid responsiveness (provided that they are interpreted properly) [32,33], we did not include these indices, since the aim was to study the value of static cardiac preload indicators. That static filling pressures were of predictive value for fluid responsiveness in our study can be explained by the low PEEP used in our patients, and this may not apply when higher PEEP is needed. Finally, predictors and monitors of fluid responsiveness were independent of the definition of the latter, even though most commonly CI responses >10% are used [33].

## Conclusions

Our study suggests that, after coronary artery and major vascular surgery, prediction and monitoring of fluid responsiveness by pressures or transpulmonary thermodilution-derived volumes depends on systolic cardiac function and the degree of cardiac dilatation. Whereas CVP may be useful for predicting fluid responsiveness in patients after coronary and major vascular surgery regardless of GEF, GEDVI is less and PAOP is more useful for predicting fluid responsiveness when GEF is low than when it is near-normal, respectively, provided that positive end-expiratory pressure is low. In practice, our data may imply use of the pulmonary artery catheter and derived filling pressures in hemodynamic monitoring of patients with impaired left ventricular systolic function and dilatation, and use of transpulmonary thermodilution and derived filling volumes in cases of relatively normal left ventricular systolic function. This may help in refining fluid therapy and preventing harmful fluid overloading.

## Key messages

• In patients after coronary artery or major vascular surgery, the relative predictive value of filling pressures and volumes for fluid responsiveness depends on left ventricular systolic function as measured by GEF

• Whereas, CVP may be useful for predicting fluid responsiveness regardless of GEF, in patients with low GEF, PAOP has a greater predictive value than GEDVI for fluid responsiveness

• In patients with near-normal GEF, GEDVI is superior to PAOP for predicting fluid responsiveness

• This study argues in favor of using pulmonary artery catheter-derived filling pressures in hemodynamic monitoring of patients with impaired left ventricular systolic function and of using transpulmonary thermodilution-derived volumes in relatively normal left ventricular systolic function

## Abbreviations

APACHE-II score: Acute Physiology and Chronic Health Evaluation II score; AUC: areas under the curve; BSA: body surface area; CI: cardiac index; CVP: central venous pressure; GEDVI: global end-diastolic volume index; GEF: global ejection fraction; HES: hydroxyethyl starch; HR: heart rate; MAP: mean arterial pressure; PAC: pulmonary artery catheter; PAOP: pulmonary artery occlusion pressure; PEEP: positive end-expiratory pressure; PiCCO: pulse contour cardiac output; ROC: receiver operating characteristic curves; SI: stroke volume index; SD: standard deviation; SVI: stroke volume index.

## Competing interests

The authors declare that they have no competing interests.

## Authors' contributions

RT analyzed and interpreted the data, and drafted and revised the article. ID, MR and AG conceived the protocol of the study, and analyzed and interpreted the data. RB facilitated the accomplishment of the study. AG drafted and revised the article. All authors gave their final approval of this version to be published.

## Supplementary Material

Additional file 1 **Supplementary Tables**. **Table S1**. Summated fluid loading responsiveness, defined as ≥10% increase in cardiac index, when global ejection fraction (GEF) is ≤15% or >15%. **Table S2**. Summated fluid loading responsiveness, defined as ≥15% increase in cardiac index, when global ejection fraction (GEF) is <20% or ≥20%. **Table S3**. Areas under the receiver operating characteristic curve (AUCs, 95% confidence intervals) for prediction of fluid responsiveness (increase in SVI ≥10% from t = 0 to 90 minutes (A) or ≥15% (B)) by baseline values at t = 0, according to global ejection fraction (GEF). Click here for file
