# Supplementary material for: Cardiac filling volumes versus pressures for predicting fluid responsiveness after cardiovascular surgery: the role of systolic cardiac function
Source: Crit Care. 2011 Feb 25;15(1):R73. doi: 10.1186/cc10062 (PMC3222006; doi:10.1186/cc10062)
Supplement: Additional file 1 — Supplementary Tables. Table S1. Summated fluid loading responsiveness, defined as ≥10% increase in cardiac index, when global ejection fraction (GEF) is ≤15% or >15%. Table S2. Summated fluid loading responsiveness, defined as ≥15% increase in cardiac index, when global ejection fraction (GEF) is <20% or ≥20%. Table S3. Areas under the receiver operating characteristic curve (AUCs, 95% confidence intervals) for prediction of fluid responsiveness (increase in SVI ≥10% from t = 0 to 90 minutes (A) or ≥15% (B)) by baseline values at t = 0, according to global ejection fraction (GEF). [file cc10062-S1.DOC]

**Additional files**

**Table S1. Summated fluid loading responsiveness, defined as ≥10% increase in cardiac index, when global ejection fraction (GEF) is ≤15% or >15%.**

|  | **GEF ≤15% (n=4)** | | | **GEF>15% (n=28)** | | |
| --- | --- | --- | --- | --- | --- | --- |
|  | Responder  (n=6 steps in 4 patients) | Non-responder  (n=6 steps in 3 patients) | P-value | Responder  (n=28 steps in 19 patients) | Non-responder  (n=56 steps in 28 patients) | P-value |
|  |  |  |  |  |  |  |
| CI, L/min/m^2^ |  |  |  |  |  |  |
| baseline | 3.0±0.7 | 3.4±0.8 | 0.068 | 3.4±0.7 | 3.8±0.8 | 0.037 |
| after | 3.6±0.7 | 3.4±0.9 |  | 4.0±0.8 | 3.8±0.7 |  |
| change | 0.6±0.3 | 0.0±0.2 | n.a. | 0.6±0.5 | 0.0±0.3 | n.a. |
|  |  |  |  |  |  |  |
| GEDVI, mL/m^2^ |  |  |  |  |  |  |
| baseline | 1531±458 | 1275±329 | 0.114 | 811±182 | 915±172 | 0.014 |
| after | 1746±587 | 1270±362 |  | 880±187 | 904±180 |  |
| change | 215±270 | -5±104 | 0.775 | 68±69 | -9±61 | 0.008 |
|  |  |  |  |  |  |  |
| CVP, mmHg |  |  |  |  |  |  |
| baseline | 5±2 | 9±2 | 0.002 | 3±2 | 6±3 | 0.042 |
| After | 6±3 | 9±2 |  | 5±2 | 7±3 |  |
| change | 1±1 | 1±1 | 0.277 | 1±1 | 1±1 | 0.975 |
|  |  |  |  |  |  |  |
| PAOP, mmHg |  |  |  |  |  |  |
| baseline | 8±3 | 12±3 | 0.039 | 8±2 | 9±3 | 0.488 |
| after | 11±2 | 14±2 |  | 10±3 | 14±4 |  |
| change | 2±2 | 2±2 | 0.639 | 1±1 | 1±2 | 0.137 |

­­Mean±SD. Abbreviations: GEF, global ejection fraction; R, responding fluid loading step (≥10% increase in CI); NR, non-responding fluid loading step; CI, cardiac index; GEDVI, global end diastolic volume index; CVP, central venous pressure; PAOP, pulmonary artery occlusion pressure: n=13, n=10, n=11 and n=21, in R and NR at GEF ≤15% and >15%, respectively; n.a., not applicable. P-values adjusted for amount and type of fluid.

**Table S2. Summated fluid loading responsiveness, defined as ≥15% increase in cardiac index, when global ejection fraction (GEF) is <20% or ≥20%.**

|  | **GEF <20% (n=12)** | | | **GEF≥20% (n=20)** | | |
| --- | --- | --- | --- | --- | --- | --- |
|  | Responder  (n=8 steps in 6 patients) | Non-responder  (n=28 steps in 10 patients) | P-value | Responder  (n=7 steps in 6 patients) | Non-responder  (n=53 steps in 20 patients) | P-value |
|  |  |  |  |  |  |  |
| CI, L/min/m^2^ |  |  |  |  |  |  |
| baseline | 3.2±0.6 | 3.5±0.9 | 0.009 | 3.1±0.5 | 3.8±0.7 | 0.024 |
| after | 3.9±0.6 | 3.7±0.9 |  | 4.0±0.9 | 3.8±0.7 |  |
| change | 0.7±0.2 | 0.2±0.2 | .n.a. | 0.9±0.8 | 0.1±0.3 | n.a. |
|  |  |  |  |  |  |  |
| GEDVI, mL/m^2^ |  |  |  |  |  |  |
| baseline | 1328±532 | 1057±227 | 0.027 | 644±104 | 869±169 | <0.001 |
| after | 1536±633 | 1070±231 |  | 730±84 | 869±178 |  |
| change | 209±228 | 13±77 | <0.001 | 85±88 | 0±60 | 0.010 |
|  |  |  |  |  |  |  |
| CVP, mmHg |  |  |  |  |  |  |
| baseline | 4±3 | 7±3 | 0.269 | 3±1 | 5±2 | 0.169 |
| After | 5±2 | 8±2 |  | 5±2 | 6±2 |  |
| change | 1±2 | 1±1 | 0.020 | 2±2 | 1±1 | 0.575 |
|  |  |  |  |  |  |  |
| PAOP, mmHg |  |  |  |  |  |  |
| baseline | 6±3 | 11±3 | <0.001 | 9±2 | 9±3 | 0.604 |
| after | 9±2 | 12±4 | . | 12±3 | 11±4 |  |
| change | 3±1 | 1±1 | 0.004 | 2±0 | 1±1 | 0.022 |
|  |  |  |  |  |  |  |
| Fluid input per step, mL | 563±106 | 468±128 | 0.025 | 549±151 | 526±111 | 0.498 |

­

Mean±SD. Abbreviations: GEF, global ejection fraction; R, responding fluid loading step (≥15% increase in CI); NR, non-responding fluid loading step; CI, cardiac index; GEDVI, global end diastolic volume index; CVP, central venous pressure; PAOP, pulmonary artery occlusion pressure, N.A., not applicable. P values adjusted for amount and type of fluid.

**Table S3. Areas under the receiver operating characteristic curve (AUC's, 95% confidence intervals) for prediction of fluid responsiveness (increase in SVI ≥10% from t=0-90 min [A] or ≥15% [B]) by baseline values at t=0, according to global ejection fraction (GEF).**

|  | **GEF <20% (n=12)** | | **GEF≥20% (n=20)** | |
| --- | --- | --- | --- | --- |
|  | AUC | P-value | AUC | P-value |
| **A** |  |  |  |  |
| GEDVI | 0.53 (0.17-0.89) | 0.865 | 0.88 (0.72-1.03) | 0.005 |
| CVP | 0.89 (0.70-1.08) | 0.034 | 0.67 (0.42-0.92) | 0.203 |
| PAOP | 1.00 (1.00-1.00) | 0.020 | 0.72 (0.45-0.99) | 0.175 |
|  |  |  |  |  |
|  |  |  |  |  |
| **B** |  |  |  |  |
| GEDVI | 0.49 (0.14-0.83) | 0.935 | 0.74 (0.52-0.96) | 0.099 |
| CVP | 0.80 (0.54-1.06) | 0.088 | 0.74 (0.53-0.96) | 0.091 |
| PAOP | 1.00 (1.00-1.00) | 0.020 | 0.75 (0.48-1.03) | 0.157 |

Abbreviations: GEF, global ejection fraction; GEDVI, global end diastolic volume index, mL/m^2^; CVP, central venous pressure, mm Hg; PAOP, pulmonary artery occlusion pressure, mm Hg.
